# Evaluating an AI Chatbot “Prostate Cancer Info” for Providing Quality Prostate Cancer Screening Information: Cross-Sectional Study

**DOI:** 10.2196/72522

**Published:** 2025-05-21

**Authors:** Otis L Owens, Michael S Leonard

**Affiliations:** 1 College of Social Work, University of South Carolina, 1512 Pendelton Street, Columbia, SC, 29208, United States, 1 8037770384

**Keywords:** generative artificial intelligence, chatbot, chatGPT, prostate cancer, cancer screening, shared decision making, artificial intelligence

## Abstract

**Background:**

Generative artificial intelligence (AI) chatbots may be useful tools for supporting shared prostate cancer (PrCA) screening decisions, but the information produced by these tools sometimes lack quality or credibility. “Prostate Cancer Info” is a custom GPT chatbot developed to provide plain-language PrCA information only from websites of key authorities on cancer and peer-reviewed literature.

**Objective:**

The objective of this paper was to evaluate the accuracy, completeness, and readability of Prostate Cancer Info’s responses to frequently asked PrCA screening questions.

**Methods:**

A total of 23 frequently asked PrCA questions were individually input into Prostate Cancer Info. Responses were recorded in Microsoft Word and reviewed by 2 raters for their accuracy and completeness. Readability of content was determined by pasting responses into a web-based Flesch Kincaid Reading Ease Scores calculator.

**Results:**

Responses to all questions were accurate and culturally appropriate. In total, 17 of the 23 questions (74%) had complete responses. The average readability of responses was 64.5 (SD 8.7; written at an 8th-grade level).

**Conclusions:**

Generative AI chatbots, such as Prostate Cancer Info, are great starting places for learning about PrCA screening and preparing men to engage in shared decision-making but should not be used as independent sources of PrCA information because key information may be omitted. Men are encouraged to use these tools to complement information received from a health care provider.

## Introduction

Generative artificial intelligence (AI) chatbots such as ChatGPT, Google Gemini, and Microsoft Copilot have become highly publicized for enhancing work efficiency and effectively responding to diverse queries. These sophisticated programs leverage large language models, machine learning, and natural language processing to understand and respond to a query with publicly available or third-party information [1]. Over the past 2 years, researchers have demonstrated a growing interest in evaluating generative AI chatbots for providing quality health and cancer information [2-4]. While the performance of generative AI chatbots has varied depending on the disease queried, complexity of the query, and brand of chatbot used, these tools show promise for being reliable health information resources in the future [3,5,6].

In terms of prostate cancer (PrCA), the second leading cause of cancer mortality among men in the United States [7], the American Cancer Society (ACS) [8], American Urological Association (AUA) [9,10], and the United States Preventive Services Task Force (USPSTF) [11] recommend that men make shared PrCA screening decisions with their health care providers. To prepare for this important decision, men need access to credible, readable, and culturally-appropriate (eg, African Americans have a higher mortality risk [12]) PrCA screening information [13]. Multiple studies have investigated the quality of PrCA information generated by AI chatbots [14-22]. Overall, these studies show that PrCA information produced by chatbots can be accurate, reliable, and moderately comprehensive, but readability and credibility are often compromised. In a recent study by the authors, Owens and Leonard [23] discovered that soliciting plain-language responses from chatbots to PrCA screening inquiries significantly enhanced the response’s readability. Conversely, credibility was difficult to ascertain because generative AI chatbots do not consistently reference authoritative information sources [23]. To create a reliable and credible plain-language resource for PrCA screening information, we have developed “Prostate Cancer Info” (PCI), a generative AI chatbot using Open AI’s custom GPT platform [24]. PCI is unique because it only responds to inquiries from credible, PrCA expert-curated websites (like the ACS). This method is different from current generative AI chatbots, which search the entire web and produce responses from a variety of expert-vetted and non-vetted sources. In addition, we have programmed PCI to always provide a source for responses, which is uncommon for current generative AI chatbots. Finally, we have programmed PCI to provide responses that do not exceed 6th to 8th grade readability as recommended by the American Medical Association [25]. The study’s purpose is to evaluate the accuracy, completeness, and readability of PCI responses to 23 frequently asked PrCA screening questions. The study will contribute insight into the safety and efficacy of using AI chatbots for shared PrCA screening decision-making and the usefulness of developing customized AI chatbots for PrCA decision-making.

## Methods

### Intervention Development

Author MSL developed PCI using a multistep process. Websites published by the ACS, AUA, USPSTF, and Centers for Disease Control and Prevention (CDC) were programmed into the GPT builder [24]. The rationale for limiting our search to these websites is that these organizations are globally recognized for providing timely, evidence-based PrCA screening education and recommendations. In particular, the PrCA screening recommendations from the ACS, AUA, and USPSTF are the most widely recognized in US PrCA research and clinical practice. PCI was then directed to draw responses exclusively from these websites in the order listed. Therefore, PCI relied on the ACS website as a primary source unless the information was unavailable or was requested from a non-ACS source. Strict directives were given to PCI to (1) only retrieve information from websites provided, (2) respond with language at or below 8th grade readability, (3) ignore non-PrCA queries, and (4) provide sources for responses. PCI was pretested to confirm its adherence to these directives.

PCI answers user questions through a well-defined process: first, it limits itself to information from preapproved websites. Then, it indexes these sites, reading and organizing their content. When a user asks a question, PCI searches its indexed data for relevant details, analyzes the information to understand the context, and creates a concise, accurate answer from approved sources. This ensures consistent and trustworthy answers.

### Study Protocol

A total of 23 frequently asked PrCA questions were adopted from previous studies by Zhu et al [15] and Owens and Leonard [23]. One author entered questions into PCI. Responses were saved in a document for rating by both authors. The authors used a coding form containing questions with key points and answers from ACS and CDC education resources[26,27], along with screening recommendations from the ACS, AUA, and USPSTF [8-11], and checkboxes to evaluate whether a response was accurate (contained correct statements) and complete (presented all salient facts without significant omissions). For example, a response to “What is the prostate?” would be considered accurate if it stated that the prostate is a gland that is a part of the male reproductive system. However, to be considered complete, the response would also need to include information on the size of the prostate, its location, and its purpose. If any parts of the response were not correct, they were rated as inaccurate. Table 1 shows the key points used to determine accuracy and completeness. Each of these key points is critical to a shared PrCA decision because a patient must consider factors such as the risks, benefits, and uncertainties of screening; their age, race, family history; and their personal values and preferences. Our chatbot responses have been included in Multimedia Appendix 1. Additional space was allotted on the coding form to record details about inaccuracies or omissions. The authors had 100% interrater agreement. The readability of responses was determined via a web-based Flesch Kincaid Reading Ease Scores calculator. Each response was copied and pasted into the calculator, excluding the reference website. The Flesch Kincaid Reading Ease Score uses total words, sentences, and syllables in an excerpt of text to calculate a score between 0 and 100, which corresponds to grade-level readability. Scores of 60 to 100 are considered easy to read by someone possessing an education at or below 8th to 9th grade. Scores of 50 to 60 require a 10th to 12th grade education (ie, fairly difficult) and scores below 50 require a college education (ie, very difficult) to comprehend.

**Table 1.** Key points for determining accuracy and completeness^a^.

Questions	Key points
Basic Questions	
What is the prostate?	Male reproductive organ. The size of the prostate increases with age but is walnut-sized in younger men. Located below the bladder and in front of the rectum. Produces some of the fluid in semen.
How common is prostate cancer?	About 313,780 new cases of prostate cancer (1 in 8 men). About 35,770 deaths from prostate cancer (1 in 44 men).
What are the risks for prostate cancer?	Risk increases with age. More common among African-American men. More prevalent in North America, northwestern Europe, Australia, and the Caribbean islands. Risk is doubled if a man has a first-degree relative (eg, father, brother, or son) with prostate cancer gene mutations can increase the risk for prostate cancer. Less common risk factors are diet, obesity, smoking, chemical exposure, inflammation of the prostate, STIs^b^, and vasectomy.
When and how often should a man be screened for prostate cancer?	ACS^c^: ages 50 years (average risk), 45 years (high risk), and 40 years (very high risk). AUA^d^: ages 45 to 50 years (average risk) and age 40 years (high risk). USPSTF^e^: age 55 to 69 years (average risk).
What are the symptoms of prostate cancer?	Can have no symptoms in early stages. Urinary problems. Blood in urine or semen. Erectile dysfunction. Pain in hips, back, chest, or other areas. Weakness or numbness in legs or feet. Loss of bladder or bowel control.
What are the types of screenings for prostate cancer?	DRE^f^ and gloved finger test are not 100% accurate. PSA^g^, a blood test, is not 100% accurate and can produce false positives and false negatives.
What are the benefits and harms of prostate cancer screening?	Benefit: can find cancer early. Harms: tests, especially PSA can produce false positives or negatives, which can lead to unnecessary tests or treatments, which carry risks.
How is prostate cancer diagnosed?	Biopsy: tissue samples from the prostate.
What are the risks of a prostate biopsy?	Pain, blood in the semen, or infection.
How long can I live if I have prostate cancer?	The 5-year relative survival rate is 97% on average, but depends on how far the cancer has spread.
Difficult Questions	
Is the PSA or DRE more effective for finding prostate cancer?	PSA is more effective.
My father had prostate cancer. Will I have prostate cancer too?	Having a father or brother with prostate cancer can more than double a man’s risk of prostate cancer.
I have a high PSA level. Do I have prostate cancer?	The probability of having prostate cancer increases with PSA level but there is no set PSA level that can definitively indicate the presence of prostate cancer.
What does a PSA level of 4 mean?	Men with a PSA level between 4 and 10 have about a 1 in 4 chance of having prostate cancer.
What does a PSA level of 10 mean?	The chance of having prostate cancer is 50% with a PSA of 10 or more.
What does a PSA level of 20 mean?	The chance of having prostate cancer is more than 50% with a PSA of 20 or more.
What newer tests for prostate cancer may be more accurate than the PSA test?	The prostate health index (PHI). 4Kscore test. IsoPSA test. Urine-based tests.
If my biopsy sample is positive for cancer, should I receive genetic testing?	Some men who have a strong family history or certain inherited genes, prior cancer diagnosis, or cancer that has spread to other parts of the body, should speak to their health care provider about this option.
If my biopsy sample is positive for cancer, how soon should I start treatment?	Will depend on the stage and grade of the cancer and their: Age and expected lifespan. Other serious health conditions. Feelings about treatment. The likelihood of a cure and doctor’s opinion. Feelings about treatment side effects.
Are there any cons to taking an at-home PSA test?	At home PSA tests do not give a man an opportunity to make a shared decision with their health care provider about the risks, benefits, and uncertainties of the PSA test.
I am an African-American male, aged 40, with a family history of prostate cancer, at what age should I begin receiving prostate cancer screening?	Screening should begin at age 40 based on both the ACS and AUA screening guidelines.
I am an African-American male, aged 40, with a family history of prostate cancer, can you provide me with all of the information I need to know to make a shared decision about prostate cancer screening?	Response should include all key points such as: Prostate cancer incidence and mortality statistics. Prostate cancer risks for African-American men. Symptoms for prostate cancer. Screenings for prostate cancer for African-American men. Risks and uncertainties of prostate cancer screening. Meaning of PSA results. Biopsy for diagnosis. Risk of biopsy. Steps after a positive biopsy.
What are the differences in screening recommendations between major health organizations?	ACS: ages 50 years (average risk), 45 years (high risk), and 40 years (very high risk). AUA: age 45 to 50 years (average risk) and age 40 years (high risk). USPSTF: age 55 to 69 years (average risk).

a Key points developed from web sources produced by ACS, CDC, AUA, and UPSTF.

b STI: sexually transmitted infection.

c ACS: American Cancer Society.

d AUA: American Urological Association.

e USPSTF: United States Preventive Services Task Force.

f DRE:digital rectal exam.

g PSA: prostate specific antigen test.

### Data Analysis

Data was transferred from coding forms to Microsoft Excel spreadsheets for analysis. Descriptive statistics were calculated to determine the percentage of questions answered accurately and completely. An average mean readability score was also calculated.

## Results

### Accuracy and Completeness

Responses to all questions were accurate. In total, 17 of 23 questions (74%) were answered completely. Of the 6 questions with less complete responses, one lacked information about geography as a risk for PrCA and the higher prevalence of PrCA in North America. Of note is that this response recognized that African Americans may be at greater risk for the disease, but statistics were not provided in any responses that substantiated the burden of incidence and mortality among African-American men. A total of 3 questions related to the meanings of PSAs of 4, 10, and 20 lacked statistics about the probability of PrCA, but did state men’s greater chance of being diagnosed with PrCA at PSAs higher than 4. A fifth question about how soon a man should start treatment after a positive biopsy lacked information about how age, expected life span, comorbidities, and patient feelings about side effects factor into treatment decisions. Finally, a sixth question about what information an African-American male, aged 40 years with a family history of PrCA needs to know to make a shared screening decision yielded an answer that lacked information about what PSA results mean or the purpose and risks of a prostate biopsy.

### Readability

The average readability was 64.5 (SD 8.7), which indicates most responses were written at an 8th-grade level or below. However, 5 of 23 responses (22%) were written at a 10th to 12th grade reading level and 1 response was written at a college level. In addition, 3 of the 5 responses addressed difficult questions. Scores ranged from 48.6 to 81.3. The lowest readability score (ie, 48.6) was in response to a basic question about symptoms of PrCA (see Table 2).

**Table 2.** Accuracy, completeness, and readability of Prostate Cancer Info responses to questions about prostate cance.

Questions	Accurate?	Complete?	Readability score
Basic questions			
What is the prostate?	Yes	Yes	81.3
How common is prostate cancer?	Yes	Yes	79.4
What are the risks for prostate cancer?	Yes	No	65.5
When and how often should a man be screened for prostate cancer?	Yes	Yes	70.3
What are the symptoms of prostate cancer?	Yes	Yes	48.6^a^
What are the types of screenings for prostate cancer?	Yes	Yes	70.8
What are the benefits and harms of prostate cancer screening?	Yes	Yes	64.6
How is prostate cancer diagnosed?	Yes	Yes	71.5
What are the risks of a prostate biopsy?	Yes	Yes	56.1^b^
How long can I live if I have prostate cancer?	Yes	Yes	63
Difficult questions			
Is the PSA^c^ or DRE^d^ more effective for finding prostate cancer?	Yes	Yes	74.7
My father had prostate cancer. Will I have prostate cancer too?	Yes	Yes	60.4
I have a high PSA level. Do I have prostate cancer?	Yes	Yes	70.7
What does a PSA level of 4 mean?	Yes	No	67.5
What does a PSA level of 10 mean?	Yes	No	67.2
What does a PSA level of 20 mean?	Yes	No	67.5
What newer tests for prostate cancer may be more accurate than the PSA test?	Yes	Yes	63.1
If my biopsy sample is positive for cancer, should I receive genetic testing?	Yes	Yes	52.4^b^
If my biopsy sample is positive for cancer, how soon should I start treatment?	Yes	No	58.8^b^
Are there any cons to taking an at-home PSA test?	Yes	Yes	65
I am an African-American male, aged 40, with a family history of prostate cancer, at what age should I begin receiving prostate cancer screening?	Yes	Yes	51.8^b^
I am an African-American male, aged 40, with a family history of prostate cancer, can you provide me with all of the information I need to know to make a shared decision about prostate cancer screening?	Yes	No	51.6^b^
What are the differences in screening recommendations between major health organizations?	Yes	Yes	60.6
Total (yes), %	100	74	—^e^
Readability score, mean (SD)	—	—	64.5 (8.7)
Readability score, median (range)	—	—	65 (48.6-81.3)

a Readability was very difficult (requires a college education).

b Readability was fairly difficult (requires a 10th to 12th grade education).

c PSA: prostate specific antigen test.

d DRE: digital rectal exam.

e Not applicable.

## Discussion

### Principal Findings

PCI had pristine accuracy and average completeness and readability. On average, completeness and readability were higher on responses to basic questions as compared to difficult questions. Specifically, 9 of 10 (90%) of the responses to basic and 8 of 13 (62%) of the responses to difficult questions were complete. In addition, 8 of 10 or (80%) and 9 of 13 (69%) of readability scores for basic and difficult questions, respectively, were below an 8th to 9th grade level. Difficult questions often contained longer and more complex responses, which likely affected readability. Furthermore, 4 of the 6 incomplete responses only lacked 1 key point which did not significantly dilute these responses. For example, 3 responses on PSA levels at 4, 10, and 20 did not effectively highlight differences in cancer likelihood (eg, over 50% chance), but each response indicated a greater chance of prostate cancer. Therefore, men would be informed that a PSA over 4 is concerning and warrants counsel from a provider. Key points missed in responses about when to start treatment for any man and about shared decision-making for African-American men are more concerning as the omitted information (eg, biopsy as a diagnosis tool) is focal to a PrCA screening decision. Not possessing this knowledge could lead to a PrCA screening decision that is not ideally informed and may not truly be shared between the patient and their healthcare provider. Specifically, knowing that the biopsy, not the PSA, is the only definitive means to diagnose PrCA may somewhat lessen the fear of an increased PSA score because another diagnostic step exists. Being informed about the biopsy could also prompt shared discussion about the relevance of a biopsy for the patient’s circumstance. In addition, providing African-American men with all the information necessary to share a PrCA screening decision based on their demographic profile could be exceptionally useful for those men who may lack access to a question list, and not have time to ask multiple questions to an AI chatbot, or simply want a more tailored answer to their given circumstance. This tailored information can also facilitate a shared PrCA screening decision that is more patient-centered.

### Limitations

The 23 questions we used may not reflect the full breadth of inquiries someone may have about PrCA screening. PCI was programmed to seek information from a finite set of websites from key medical authorities, but several equally credible websites were not included (eg, Mayo Clinic), which may have slightly improved PCI’s performance. While much of the general information about PrCA on these additional websites (eg, signs, symptoms, and prostate anatomy) would likely be similar, there may be cutting-edge research on new PrCA screenings that may not yet be publicized on ACS or similar websites but could provide additional context for more difficult questions like those related to newer tests for PrCA. Finally, although rigorous research methods, such as interrater reliability, were used to mitigate any study bias, we acknowledge that as the developers of PCI, we may be susceptible to unconscious biases that could have affected our ratings. For transparency, we have included all PCI responses in Multimedia Appendix 1. Future studies to evaluate PCI and similar chatbots should include external raters and user feedback.

### Comparison With Previous Work

Similar to our previous research [23] and research by others [15-19,21,22], generative AI chatbots like PCI can be highly accurate when responding to PrCA and PrCA screening inquiries. The completeness and readability of Prostate Info’s responses to PrCA screening questions varied. PCI generally performed better than Lombardo et al [20] and comparable with Geantă et al [20-22], both of whom investigated chatbot performance on non-US PrCA guidelines. As compared with studies using US PrCA guidelines, PCI performed better than ChatGPT-3.5 (OpenAI), ChatGPT-4 (OpenAI), Microsoft Co-Pilot, Google Gemini, and Google Gemini Advanced, but equal to Microsoft Copilot on completeness of responses to basic PrCA screening queries posed in our previous comparative study, which solicited both standard and plain-language (ie low literacy) responses [23]. However, the average readability on basic questions was lower than all, but one (ie, Microsoft Co-Pilot) generative AI chatbot when considering plain language responses only [23]. Otherwise, PCI outperformed all, but one (ie, Google Gemini Advanced) chatbot when we asked it to provide a standard response [23]. Compared to Zhu et al [15], who evaluated multiple generative AI chatbots’ performance on a similar combination of basic and difficult questions to our study, PCI did not perform nearly as well as ChatGPT and ChatGPT Plus (earlier versions of ChatGPT) on completeness. PCI outperformed all other chatbots evaluated by Zhu et al [15] including Perplexity (by Perplexity AI), YouChat (by You.com), Chatsonic (by Writesonic), and NeevaAI (by Neeva). However, PCI’s average readability may have been (but not definitively) lower than all generative AI chatbots evaluated in Zhu and colleagues’ [15] study. It is important to note that Zhu et al [15] used a slightly different method for calculating completeness and readability than this study or the study by Owens and Leonard [23]. Zhu et al [15] determined the percentage of comprehensiveness using a Likert approach as opposed to indicating whether a response was simply complete or not complete. Numbers listed in Table 3 for Zhu et al [15] represent the percentage of questions that were “very comprehensive” (ie, fully complete). Readability was rated by reviewers as opposed to using a validated readability measure. Percentages reported in Table 3 for Zhu et al [15], represent that percentage of total responses that were “very easy to read.” SDs for Zhu et al [15] were not reported.

**Table 3.** Comparison of completeness and readability of chatbot responses on US prostate cancer screening guidelines.

Study	Chatbot name	Completeness, n/N (%)	Average readability score	
			mean (SD)	%, mean (SD)
This study	PCI^a^	17/23 (74)	64.5 (8.7)	—^b^
Zhu et al [15]	ChatGPT	21/22 (95)^c^	—	100 (NR^d^)
Zhu et al [15]	ChatGPT Plus	20.3/22 (92)^c^	—	100 (NR)
Zhu et al [15]	ChatSonic	14.3/22 (65)	—	95 (NR)
Zhu et al [15]	YouChat	10.34/22 (47)	—	98 (NR)
Zhu et al [15]	Neeva AI	8.8/22 (40)	—	84 (NR)
Zhu et al [15]	Perplexity Detailed	6.6/22 (30)	—	95 (NR)
Zhu et al [15]	Perplexity Concise	6.6/22 (30)	—	95 (NR)
Owens et al [23]	ChatGPT 3.5 standard response	6/11 (54)	38.0 (7.6)	—
Owens et al [23]	ChatGPT 3.5 low literacy response	4/11 (34)	70.3 (7.2)^e^	—
Owens et al [23]	ChatGPT 4.0 standard response	7/11 (63)	43.1 (9.2)	—
Owens et al [23]	ChatGPT 4.0 low literacy response	7/11 (63)	74.1 (9.9)^e^	—
Owens et al [23]	Google Gemini standard response	6/11 (54)	55.7 (10.4)	—
Owens et al [23]	Google Gemini low literacy response	5/11 (45)	81.0 (3.6)^e^	—
Owens et al [23]	Google Gemini Advanced standard response	6/11 (54)	66.3 (9.4)^e^	—
Owens et al [23]	Google Gemini Advanced low literacy response	6/11 (54)	79.4 (5.1)^e^	—
Owens et al [23]	Microsoft Copilot standard response	8/11 (72)	50.8 (9.3)	—
Owens et al [23]	Microsoft Copilot low literacy response	6/11 (54)	65.1 (6.6)^e^	—
Owens et al [23]	Microsoft Copilot Pro standard response	7/11 (63)	61.2 (9.5)	—
Owens et al [23]	Microsoft Copilot Pro low literacy response	6/1 (54)	78.8 (4.7)^e^	—

a PCI: Prostate Cancer Info.

b Not applicable.

c Chatbot had a higher completeness score than PCI.

d NR: not reported.

e Chatbot had definitively higher readability scores than PCI based on the Flesch-Kincaid readability. Other scores may also be higher but were not based on a validated measure.

We expected PCI to outperform most other commercially available generative AI chatbots on completeness because of its development using the latest ChatGPT-4.0 technology and its directive to secure information from specific websites, but PCI underperformed earlier versions of ChatGPT. Therefore, additional training of the large language model that undergirds ChatGPT-4.0 will be needed for this niche area. In addition, unexpected was the lower average readability of responses from PCI, especially compared to our previous work, which solicited plain-language responses from multiple chatbots including ChatGPT-3.5 and ChatGPT-4. Nonetheless, the average readability of PCI is suitable for an audience with a middle school education.

### Conclusions and Future Directions

Generative AI chatbots, such as PCI, are great starting places for learning about PrCA screening and preparing for shared decision-making but should not yet be used as sole sources of PrCA information because of their periodic omission of key information. Nevertheless, with further testing and validation, model training, and refinement of the source selection process, we hope PCI can be a publicly available resource for credible, evidence-based, and culturally appropriate information for PrCA screening decisions. In the future, PCI could be integrated into the decision-making workflow by prompting patients to use PCI before their medical visit via an emailed link or a 1-page hard copy with a QR code. This same email or document could contain multiple frequently asked questions about PrCA. Men should be encouraged to pose as many of these questions as possible, but especially those on our list that are more complex (eg, Is the PSA or DRE more effective for finding prostate cancer?) PCI questions and responses could then be saved on their mobile device or printed, notated to indicate areas of concern or need for clarity, and then taken to their appointment to be used to guide the shared PrCA screening discussion. During this discussion, the health care provider should ensure that men understand their personal PrCA risk; screening options; and risks, benefits, and uncertainties of PrCA screening. The discussion should then shift to focus on men’s questions and their screening preferences. Using this method of generative AI integration into the shared decision process could fortify men’s PrCA knowledge and identify patient values and preferences.

To improve the overall performance of PCI in the future, it will be necessary to iteratively fine-tune our model which will include expanding the sources from which PCI retrieves data, which could include the most current peer-reviewed journal articles in addition to websites of major research hospitals and international health organizations. All sources will be curated by a PrCA expert who will review each data source to ensure it contains quality information. Equally important will be soliciting routine feedback from health care providers and patients through an embedded satisfaction survey that can enable them to comment on the quality of questions developed by the research team, potential questions that should be added to the database, and the quality of the responses generated by the PCI. Additional model training will come from tracking common follow-up questions from users to incorporate them into the initial responses. Based on these continuous feedback loops, PCI’s performance could be improved significantly and always remain up-to-date. Future research should focus on the clinical deployment of PCI and testing to assess its acceptability, ease of use in a clinical workflow, and usefulness in the shared PrCA screening decision process.

## Supplementary material

10.2196/72522 Multimedia Appendix 1 Generative artificial intelligence (AI) chatbot responses.

## Abbreviations

ACS: American Cancer Society
AI: artificial intelligence
AUA: American Urological Association
CDC: Centers for Disease Control and Prevention
PCI: Prostate Cancer Info
PrCA: prostate cancer
USPSTF: United States Preventive Services Task Force

## References

[1] How ChatGPT and our foundation models are developed. Open AI.

[2] Milne-Ives M, de Cock C, Lim E (2020). The effectiveness of artificial intelligence conversational agents in health care: systematic review. J Med Internet Res.

[3] Wang L, Wan Z, Ni C (2024). A systematic review of ChatGPT and other conversational large language models in healthcare. medRxiv.

[4] Xu L, Sanders L, Li K, Chow JCL (2021). Chatbot for health care and oncology applications using artificial intelligence and machine learning: systematic review. JMIR Cancer.

[5] Potapenko I, Boberg-Ans LC, Stormly Hansen M, Klefter ON, van Dijk EHC, Subhi Y (2023). Artificial intelligence-based chatbot patient information on common retinal diseases using ChatGPT. Acta Ophthalmol.

[6] Shuaib A (2024). Transforming healthcare with AI: promises, pitfalls, and pathways forward. Int J Gen Med.

[7] Siegel RL, Giaquinto AN, Jemal A (2024). Cancer statistics, 2024. CA Cancer J Clin.

[8] Wolf AMD, Wender RC, Etzioni RB (2010). American Cancer Society guideline for the early detection of prostate cancer: update 2010. CA Cancer J Clin.

[9] Wei JT, Barocas D, Carlsson S (2023). Early detection of prostate cancer: AUA/SUO guideline Part I: prostate cancer screening. J Urol.

[10] Wei JT, Barocas D, Carlsson S (2023). Early detection of prostate cancer: AUA/SUO guideline part II: considerations for a prostate biopsy. J Urol.

[11] Curry SJ, Owens DK, Domingo KB, US Preventive Services Task Force (2018). Screening for prostate cancer: US Preventive Services Task Force recommendation statement. JAMA.

[12] Giaquinto AN, Miller KD, Tossas KY, Winn RA, Jemal A, Siegel RL (2022). Cancer statistics for African American/Black People 2022. CA A Cancer J Clinicians.

[13] Grene M, Cleary Y, Marcus-Quinn A (2017). Use of plain-language guidelines to promote health literacy. IEEE Trans Profess Commun.

[14] Coskun B, Ocakoglu G, Yetemen M, Kaygisiz O (2023). Can ChatGPT, an artificial intelligence language model, provide accurate and high-quality patient information on prostate cancer?. Urology.

[15] Zhu L, Mou W, Chen R (2023). Can the ChatGPT and other large language models with internet-connected database solve the questions and concerns of patient with prostate cancer and help democratize medical knowledge?. J Transl Med.

[16] Alasker A, Alsalamah S, Alshathri N (2024). Performance of large language models (LLMs) in providing prostate cancer information. BMC Urol.

[17] Musheyev D, Pan A, Loeb S, Kabarriti AE (2024). How well do artificial intelligence chatbots respond to the top search queries about urological malignancies?. Eur Urol.

[18] Gibson D, Jackson S, Shanmugasundaram R (2024). Evaluating the Efficacy of ChatGPT as a Patient Education Tool in Prostate Cancer: Multimetric Assessment. J Med Internet Res.

[19] Chiarelli G, Stephens A, Finati M (2024). Adequacy of prostate cancer prevention and screening recommendations provided by an artificial intelligence-powered large language model. Int Urol Nephrol.

[20] Lombardo R, Gallo G, Stira J (2025). Quality of information and appropriateness of Open AI outputs for prostate cancer. Prostate Cancer Prostatic Dis.

[21] Geantă M, Bădescu D, Chirca N (2024). The emerging role of large language models in improving prostate cancer literacy. Bioengineering (Basel).

[22] Geantă M, Bădescu D, Chirca N (2024). The potential impact of large language models on doctor-patient communication: a case study in prostate cancer. Healthcare (Basel).

[23] Owens OL, Leonard M (2025). A comparison of prostate cancer screening information quality on standard and advanced versions of chatgpt, google gemini, and microsoft copilot: a cross-sectional study. Am J Health Promot.

[24] GPT builder. Open AI.

[25] Weiss BD (2007). Health Literacy and Patient Safety: Help Patients Understand Manual for Clinicians.

[26] (2025). Prostate cancer. American Cancer Society.

[27] (2024). Prostate cancer. Centers for Disease Control and Prevention.

