# Supplementary material for: Evaluating an AI Chatbot “Prostate Cancer Info” for Providing Quality Prostate Cancer Screening Information: Cross-Sectional Study
Source: JMIR Cancer. 2025 May 21;11:e72522. doi: 10.2196/72522 (PMC12118940; doi:10.2196/72522)
Supplement: Multimedia Appendix 1 [file cancer-v11-e72522-s001.docx]

**Supplement 1: Prostate Cancer Info Responses to PrCA Screening Inquiries**

**1. What is the prostate?**

The prostate is a small gland in the male body. It is about the size of a walnut and is located below the bladder and in front of the rectum. The prostate's main job is to help make semen, the fluid that carries sperm. It surrounds part of the urethra, the tube that carries urine and semen out of the body.

**2. How common is prostate cancer?**

Prostate cancer is the most common cancer in men in the U.S. (after skin cancer). In 2025, about 313,780 men are expected to be diagnosed with it. That means about 1 in 8 men will get prostate cancer at some point in their lives.

The risk increases with age, and it's most common in men over 65. It's also more common in African American men than in men of other races.

The good news is that most men diagnosed with prostate cancer do not die from it. More than 3.3 million men in the U.S. who had prostate cancer are still alive today.

**3. What are the risks for prostate cancer?**

The biggest risk factors for prostate cancer are:

1. **Age:** Risk goes up as you get older. Most cases happen in men over 65.
2. **Family History:** If your father, brother, or son had prostate cancer, your risk doubles. The risk is even higher if multiple family members had it, especially at a young age.
3. **Race:** African American men have the highest risk. They are also more likely to get aggressive prostate cancer. Asian American and Hispanic men have a lower risk.
4. **Genetics:** Some inherited gene changes (like BRCA1 & BRCA2) increase risk. Men with Lynch syndrome also have a higher chance.
5. **Diet & Lifestyle:** Eating a lot of red meat or high-fat dairy may raise risk. Not eating enough fruits & vegetables could also play a role. Obesity may lead to more aggressive prostate cancer.
6. **Chemical Exposure:** Some studies suggest exposure to Agent Orange (used in Vietnam War) could increase risk.

**4. When and how often should a man be screened for prostate cancer?**

The American Cancer Society (ACS) recommends that men talk to their doctor about prostate cancer screening based on their age and risk level:

1. **Age 50 – Men at Average Risk:** If you have no family history and are not in a high-risk group, talk to your doctor at age 50.
2. **Age 45 – Men at Higher Risk:** If you are African American OR if your father, brother, or son had prostate cancer before age 65, talk to your doctor at 45.
3. **Age 40 – Men at the Highest Risk:** If you have more than one close relative (father, brother, son) who had prostate cancer at an early age, talk to your doctor at 40.

**How Often Should Screening Happen?**

- If your PSA (prostate-specific antigen) test is low, you may only need testing every 2 years.
- If your PSA level is higher, your doctor may recommend yearly testing.

**Types of Screening Tests:**

1. PSA Blood Test – Measures the level of PSA in your blood.
2. Digital Rectal Exam (DRE) – The doctor checks your prostate for lumps.

**Important Note:**

- Screening is a choice – Talk with your doctor about the benefits and risks before deciding.
- Some prostate cancers grow slowly and never cause problems, so not all men need treatment right away.

**5. What are the symptoms of prostate cancer?**

In the early stages, prostate cancer often has no symptoms. That’s why screening is important. But when symptoms do appear, they may include:

**Urinary Problems:**

- Trouble starting or stopping urine flow
- Weak or slow urine stream
- Frequent urination, especially at night
- Feeling like you can’t empty your bladder
- Pain or burning when urinating (less common)

**Sexual Problems:**

- Trouble getting or keeping an erection (ED)
- Less semen when ejaculating
- Pain during ejaculation

**Other Possible Symptoms:**

- Blood in urine or semen
- Pain in the lower back, hips, or pelvis (if cancer has spread)
- Unexplained weight loss (in advanced cases)

**Important to Know:**

- Other conditions (like an enlarged prostate or infection) can cause similar symptoms.
- If you notice any of these signs, talk to a doctor.

For more details, visit [cancer.org](http://www.cancer.org).

**6. What are the types of screenings for prostate cancer?**

There are two main tests to check for prostate cancer:

1. **PSA Blood Test:** Measures the level of prostate-specific antigen (PSA) in your blood. Higher PSA levels may mean prostate cancer, but other conditions (like an enlarged prostate or infection) can also raise PSA. If PSA is high, your doctor may suggest more tests.
2. **Digital Rectal Exam (DRE):** The doctor feels the prostate with a gloved finger through the rectum. Checks for lumps, hardness, or irregular shape. Not as common as PSA but may be used with it.

**What Happens if Results Are Abnormal?**

- Your doctor may repeat the PSA test to confirm results.
- A prostate biopsy (small tissue sample) may be done to check for cancer.
- Other tests like an MRI may help decide if a biopsy is needed.

**Should You Get Screened?**

- Talk to your doctor about the benefits and risks before deciding.
- Screening is not for everyone, since some prostate cancers grow slowly and may not need treatment.

**7. What are the benefits and harms of prostate cancer screening?**

**Benefits of Screening:**

1. Finds Cancer Early: Screening can detect prostate cancer before symptoms appear. Early-stage cancer is easier to treat and may have better outcomes.
2. May Reduce the Risk of Death: Some studies show that PSA testing lowers the risk of dying from prostate cancer, especially for high-risk men.
3. More Treatment Options: If cancer is caught early, less aggressive treatments may be an option.

**Harms of Screening:**

1. False Positives (Unnecessary Worry & Tests): A high PSA level doesn’t always mean cancer. This can lead to unnecessary biopsies, which can be uncomfortable and cause infection or bleeding.
2. Overdiagnosis & Overtreatment: Some prostate cancers grow so slowly they never cause harm. But if found, doctors may still recommend treatment, which can cause serious side effects (like erectile dysfunction and urinary problems).
3. Side Effects from Treatment: Surgery and radiation can cause incontinence (trouble controlling urine) and erectile dysfunction. Some treatments can also cause bowel problems.
4. False Negatives: A normal PSA doesn’t always mean you’re cancer-free. Some prostate cancers don’t raise PSA much, so they could be missed.

**Should You Get Screened?**

- Screening is a choice – talk to your doctor about the risks and benefits.
- Men at higher risk (African American men or those with a family history) may benefit more from screening.

**8. How is prostate cancer diagnosed?**

If your doctor thinks you might have prostate cancer, they will do tests to check. The main steps are:

1. PSA Blood Test: Measures prostate-specific antigen (PSA) in your blood. High PSA may mean cancer, but other conditions (like an enlarged prostate) can also raise PSA levels.
2. Digital Rectal Exam (DRE): The doctor feels the prostate through the rectum to check for lumps or hardness.
3. Prostate Biopsy (Only If Needed): If PSA or DRE results are abnormal, a biopsy is done. A small sample of prostate tissue is taken with a needle and checked for cancer.

**Other Tests That May Help:**

- MRI or Ultrasound – Helps doctors see if a biopsy is needed.
- Gleason Score – If cancer is found, this test shows how aggressive it is.
- Bone Scan, CT Scan, or MRI – Checks if cancer has spread (only done in some cases).

**What Happens Next?**

- If cancer is found, your doctor will explain the stage and treatment options.
- Some prostate cancers grow slowly, so some men may choose active surveillance instead of treatment right away.

For more details, visit [cancer.org](http://www.cancer.org).

**9. What are the risks of a prostate biopsy?**

**Risks of a Prostate Biopsy:**

A prostate biopsy is generally safe, but like any medical procedure, it has some risks.

1. Bleeding: Blood in urine for a few days. Blood in semen (can last a few weeks). Rectal bleeding (if the biopsy is done through the rectum).
2. Infection: Some men develop a urinary tract infection (UTI) or prostate infection (prostatitis). In rare cases, a serious infection (sepsis) can happen. Antibiotics are usually given before the biopsy to lower infection risk.
3. Pain or Discomfort: Some men feel soreness in the biopsy area. Pain is usually mild and goes away in a few days.
4. Trouble Urinating: Some men may have trouble urinating after the biopsy. In rare cases, a temporary catheter may be needed.

**Should You Be Worried?**

- Most men recover quickly from a biopsy with no serious problems.
- Tell your doctor if you have fever, chills, or trouble urinating after a biopsy—this could be a sign of infection.

For more details, visit [cancer.org](http://www.cancer.org).

**10. How long can I live if I have prostate cancer?**

How long someone can live with prostate cancer depends on many things, like the stage of the cancer, how fast it is growing, and overall health.

The good news is that most men with prostate cancer live a long time. According to the American Cancer Society, the 5-year survival rate for prostate cancer is nearly 100% when the cancer is found early and has not spread far. Even if the cancer has spread to nearby areas, the survival rate is still high. However, if the cancer spreads to distant parts of the body, the survival rate drops to about 32%.

Many men with prostate cancer live for many years, especially if the cancer grows slowly. Some men die with prostate cancer rather than from it.

To get the best outcome, it's important to talk with your doctor about treatment options and regular check-ups.

For more details, you can visit the American Cancer Society: [cancer.org](http://www.cancer.org).

**11. Is the PSA or DRE more effective for finding prostate cancer?**

The PSA (Prostate-Specific Antigen) test is generally more effective than the DRE (Digital Rectal Exam) for finding prostate cancer early.

PSA Test: This is a blood test that checks for PSA, a protein made by the prostate. Higher PSA levels can be a sign of cancer, but they can also be caused by other things like an enlarged prostate or infection.

**12. My father had prostate cancer. Will I have prostate cancer too?**

Having a father with prostate cancer does not mean you will definitely get it, but it does increase your risk.

According to the American Cancer Society, if your father or brother had prostate cancer, your risk doubles. If multiple family members had it, especially at a young age, your risk is even higher. This is because some prostate cancers run in families due to inherited genes.

**What You Can Do:**

- Talk to your doctor about your risk.
- Consider PSA testing earlier (around age 40-45) if you have a strong family history.
- Stay healthy by eating well, exercising, and maintaining a healthy weight.

Having a family history is just one risk factor—many men with family history never get prostate cancer.

For more details, visit the American Cancer Society: [www.cancer.org](https://emailsc-my.sharepoint.com/personal/owenso_mailbox_sc_edu/Documents/Documents/Manuscripts/2025/Well%20Words/Revision/www.cancer.org).

**13. I have a high PSA level. Do I have prostate cancer?**

A high PSA level does not always mean you have prostate cancer. There are other reasons why PSA can be high, such as:

- Enlarged prostate (BPH) – a common condition as men age
- Prostate infection (prostatitis)
- Recent ejaculation or certain medical procedures
- Older age

Some men with high PSA do not have cancer, and some men with low PSA do have cancer. That’s why doctors look at trends over time and may do more tests, like a biopsy, if needed.

If your PSA is high, talk to your doctor about the next steps. They may recommend watching it over time or doing other tests to find out more.

For more details, visit the American Cancer Society: [www.cancer.org](https://emailsc-my.sharepoint.com/personal/owenso_mailbox_sc_edu/Documents/Documents/Manuscripts/2025/Well%20Words/Revision/www.cancer.org)..

**14. What does a PSA level of 4 mean?**

A PSA level of 4 ng/mL is often used as a cutoff for deciding if more testing is needed. However, it does not automatically mean you have prostate cancer.

**What a PSA Level of 4 Can Mean:**

- Many doctors consider PSA under 4 to be normal.
- PSA between 4 and 10 could mean a higher risk of prostate cancer, but it can also be caused by other conditions like an enlarged prostate (BPH) or an infection.
- PSA over 10 increases the chance of cancer, but further testing (like a biopsy) is needed to confirm.

Doctors also look at PSA trends over time, not just one test result. If your PSA is 4, your doctor may recommend watching it closely or doing more tests.

For more details, visit the American Cancer Society: [www.cancer.org](https://emailsc-my.sharepoint.com/personal/owenso_mailbox_sc_edu/Documents/Documents/Manuscripts/2025/Well%20Words/Revision/www.cancer.org)..

**15. What does a PSA level of 10 mean?**

A PSA level of 10 ng/mL or higher means there is a greater chance of prostate cancer, but it does not guarantee that you have cancer. Other conditions, like an enlarged prostate (BPH) or infection, can also raise PSA levels.

**What a PSA Level of 10 Might Mean:**

- Higher cancer risk – Studies show that men with a PSA over 10 have a greater chance of having prostate cancer.
- Other causes – Infections or an enlarged prostate can also increase PSA.
- Next steps – Your doctor may suggest more tests, like a repeat PSA test, MRI, or biopsy, to find out the cause.

Doctors look at PSA trends over time rather than just one test result. If your PSA is high, talk to your doctor about the next steps.

For more details, visit the American Cancer Society: [www.cancer.org](https://emailsc-my.sharepoint.com/personal/owenso_mailbox_sc_edu/Documents/Documents/Manuscripts/2025/Well%20Words/Revision/www.cancer.org)..

**16. What does a PSA level of 20 mean?**

A PSA level of 20 ng/mL or higher can be a warning sign, but it does not always mean cancer. Other conditions, like an enlarged prostate (BPH) or infection (prostatitis), can also raise PSA levels.

**What a PSA Level of 20 Might Mean:**

- Higher chance of prostate cancer – The risk is greater when PSA is this high, but more tests are needed to confirm.
- Other possible causes – A large prostate, recent procedures, or infections can also raise PSA.
- Next steps – Your doctor may suggest a repeat PSA test, imaging (MRI), or a biopsy to check for cancer.

Doctors also look at PSA changes over time to decide the best approach. If your PSA is 20, it's important to follow up with your doctor.

For more details, visit the American Cancer Society: [www.cancer.org](https://emailsc-my.sharepoint.com/personal/owenso_mailbox_sc_edu/Documents/Documents/Manuscripts/2025/Well%20Words/Revision/www.cancer.org).

**17. What newer tests for prostate cancer may be more accurate than the PSA test?**

Newer tests may help find prostate cancer more accurately than the PSA test alone. These tests can reduce unnecessary biopsies and detect aggressive cancers earlier.

**Newer Prostate Cancer Tests:**

1. Free PSA Test (fPSA) – Measures different types of PSA to see if a high PSA is more likely from cancer or another cause.
2. 4Kscore Test – Combines PSA, other blood markers, and clinical data to estimate cancer risk.
3. Prostate Health Index (PHI) – Uses a formula with different PSA types to improve accuracy.
4. PCA3 Test – A urine test that looks for a prostate cancer-related gene.
5. MRI or MRI Fusion Biopsy – Advanced imaging to better target biopsies if cancer is suspected.

Doctors may use these tests along with PSA to decide if more testing, like a biopsy, is needed.

For more details, visit the American Cancer Society: [www.cancer.org](https://emailsc-my.sharepoint.com/personal/owenso_mailbox_sc_edu/Documents/Documents/Manuscripts/2025/Well%20Words/Revision/www.cancer.org).

**18. If my biopsy sample is positive for cancer, should I receive genetic testing?**

If your biopsy is positive for prostate cancer, genetic testing may be helpful, but it depends on your personal and family history.

**Who Should Consider Genetic Testing?**

According to the American Cancer Society, you might benefit from genetic testing if:

- You have a family history of prostate, breast, ovarian, or pancreatic cancer.
- You were diagnosed with aggressive or advanced prostate cancer (like metastatic or high-grade cancer).
- You have a known genetic mutation in your family (such as BRCA1, BRCA2, or Lynch syndrome).

**Why Get Genetic Testing?**

- It can help guide treatment—some treatments work better for men with certain gene changes.
- It can show if family members may also be at higher risk.

If you're unsure, talk to your doctor or a genetic counselor to see if testing is right for you.

For more details, visit the American Cancer Society: [www.cancer.org](https://emailsc-my.sharepoint.com/personal/owenso_mailbox_sc_edu/Documents/Documents/Manuscripts/2025/Well%20Words/Revision/www.cancer.org).

**19. If my biopsy sample is positive for cancer, how soon should I start treatment?**

If your biopsy is positive for prostate cancer, the timing of treatment depends on several factors, including how aggressive the cancer is and your overall health.

**When to Start Treatment?**

- Low-risk cancer (slow-growing, low PSA, low Gleason score) – You may not need treatment right away. Many men choose active surveillance, which means regular check-ups and tests to watch the cancer.
- Intermediate- or high-risk cancer – If the cancer is growing faster or has a higher Gleason score, treatment may need to start sooner (within weeks to a few months).
- Aggressive or advanced cancer – If the cancer is spreading, treatment should start as soon as possible to slow its growth.

**Next Steps:**

- Talk to your doctor about your Gleason score, PSA level, and cancer stage.
- Discuss all treatment options, including surgery, radiation, hormone therapy, or active surveillance.
- Take time to understand your choices, but don’t wait too long if the cancer is aggressive.

For more details, visit the American Cancer Society: [www.cancer.org](https://emailsc-my.sharepoint.com/personal/owenso_mailbox_sc_edu/Documents/Documents/Manuscripts/2025/Well%20Words/Revision/www.cancer.org).

**20. Are there any cons to taking an at-home PSA test?**

Yes, there are some downsides to taking an at-home PSA (prostate-specific antigen) test.

**Accuracy Concerns** – At-home tests may not be as accurate as tests done in a medical lab. The results could be affected by how the sample is collected, stored, or tested.

**False Positives or Negatives** – A PSA test alone cannot diagnose prostate cancer. A high PSA level doesn’t always mean cancer, and a normal PSA level doesn’t always mean you’re cancer-free. This could cause unnecessary worry or false reassurance.

**No Doctor Guidance** – If your PSA level is high, you won’t have a doctor there to explain what it means or what to do next. You may need to see a doctor anyway for follow-up testing.

**Misses Other Factors** – A PSA test is just one part of prostate cancer screening. A doctor also considers age, family history, and other risk factors.

If you’re thinking about taking an at-home PSA test, it’s a good idea to talk to your doctor first. They can help you understand the pros and cons and decide what’s best for you.

For more information, check out the American Cancer Society.

**21. I am an African-American male, aged 40, with a family history of prostate cancer, at what age should I begin receiving prostate cancer screening?**

Because you are African American and have a family history of prostate cancer, you are at higher risk for the disease. The American Cancer Society recommends that men at higher risk start talking to their doctor about prostate cancer screening at age 45.

However, for men at even higher risk—such as those with more than one close family member (father, brother) diagnosed at an early age—the discussion should start at age 40. Since you have a family history, it would be a good idea to talk to your doctor now about whether you should begin screening.

Prostate cancer screening usually includes a PSA (prostate-specific antigen) blood test, and in some cases, a digital rectal exam (DRE). Your doctor can help decide the best approach for you.

For more details, visit the American Cancer Society.

**22. I am an African-American male, aged 40, with a family history of prostate cancer, can you provide me with all of the information I need to know to make an informed decision about prostate cancer screening?**

I understand you're seeking comprehensive information to make an informed decision about prostate cancer screening, given your background as a 40-year-old African American man with a family history of the disease. Here's what you need to know:

**Risk Factors:**

- Race: African American men are more likely to develop prostate cancer and often at a younger age. [cancer.org](http://www.cancer.org)
- Family History: Having a father or brother with prostate cancer more than doubles your risk. [cancer.org](http://www.cancer.org)

**Screening Recommendations:**

- Age to Start Discussion: The American Cancer Society suggests that men at high risk, including African American men and those with a first-degree relative diagnosed before age 65, begin discussing screening with their doctor at age 45. For those with more than one first-degree relative diagnosed at an early age, it's recommended to start this discussion at age 40. [cancer.org](http://www.cancer.org)

**Screening Tests:**

- Prostate-Specific Antigen (PSA) Test: A blood test measuring PSA levels, which can be higher in men with prostate cancer.
- Digital Rectal Exam (DRE): A physical exam where a doctor feels the prostate gland through the rectum to check for abnormalities.

**Potential Benefits of Screening:**

- Detecting high-risk cancers early, which can lead to timely treatment.
- Providing peace of mind if results are normal.

**Potential Harms of Screening:**

- False Positives: Elevated PSA levels can occur due to non-cancerous conditions, leading to unnecessary worry and additional tests.
- Overdiagnosis: Some prostate cancers grow slowly and may not cause problems, but detecting them could lead to treatments with side effects.
- Side Effects of Treatment: Treatments can have side effects, so it's important to weigh the benefits and risks.

**Making an Informed Decision:**

It's essential to have a detailed discussion with your healthcare provider about the pros and cons of screening. Consider your personal risk factors, overall health, and preferences. Your doctor can help you understand the potential outcomes and guide you in making the best choice for your situation.

For more detailed information, you can visit the American Cancer Society's guidelines on prostate cancer screening and the Centers for Disease Control and Prevention's resources on prostate cancer screening.

**Sources**

**23. What are the differences in screening recommendations between major health organizations?**

Different health organizations have varying recommendations for prostate cancer screening. Here's a simple breakdown:

**American Cancer Society (ACS):**

- Men should talk with their doctor about the pros and cons of prostate cancer screening.
- This discussion should start at age 50 for men at average risk.
- Men at higher risk, such as African American men and those with a close family member who had prostate cancer before age 65, should have this talk starting at age 45.
- Men with more than one close family member who had prostate cancer before age 65 should start discussing screening at age 40.
- If a man's PSA level is less than 2.5 ng/mL, he may only need to be retested every 2 years.
- If the PSA level is 2.5 ng/mL or higher, testing should be done yearly.
- Men who aren't expected to live at least 10 more years shouldn't be offered screening, as they aren't likely to benefit. [cancer.org](http://www.cancer.org)

**Centers for Disease Control and Prevention (CDC):**

- Men aged 55 to 69 should make individual decisions about being screened for prostate cancer.
- Before deciding, men should talk to their doctor about the benefits and harms of screening.
- Men aged 70 and older should not be routinely screened. [cdc.gov](http://www.cdc.gov)

**American Urological Association (AUA):**

- Men aged 55 to 69 should discuss the benefits and risks of screening with their doctor and make a shared decision.
- For men at higher risk, such as those with Black ancestry, certain genetic mutations, or a strong family history of prostate cancer, screening may start between ages 40 to 45.
- To reduce potential harms, it's suggested to have screening intervals of two years or more. [auanet.org](http://www.aua.net)

**U.S. Preventive Services Task Force (USPSTF):**

- Men aged 55 to 69 should make an individual decision about being screened after a conversation with their doctor about the potential benefits and harms.
- Men aged 70 and older are advised against routine screening. [uspreventiveservicestaskforce.org](http://uspreventiveservicestaskforce.org)

In summary, while recommendations vary slightly, all organizations emphasize the importance of discussing screening options with a healthcare provider to make an informed decision based on individual risk factors and health status.
